# Supplementary material for: Fasting plasma glucose and serum 25-hydroxy vitamin D levels in individuals with Middle Eastern and Swedish descent
Source: J Diabetes Metab Disord. 2023 May 22;22(2):1159–65. doi: 10.1007/s40200-023-01226-0 (PMC10201497; doi:10.1007/s40200-023-01226-0)
Supplement: Supplementary file 1 — Supplementary Material 1 [file 40200_2023_1226_MOESM1_ESM.docx]

Fasting Plasma Glucose and Serum 25-hydroxy Vitamin D Levels in Individuals with Middle Eastern and Swedish Descent.

Marina Taloyan ^1,2^, Kristin Hjörleifdottir Steiner ^2^, Claes-Göran Östenson ^3^, Helena Salminen ^1,2^

^1^Division of Family Medicine and Primary Care, Department of Neurobiology, Department of Neurobiology, Care sciences and Society, Karolinska Institutet, Alfred Nobels allé 23, S-141 83 Huddinge, Sweden

^2^Academic Primary Health Care Centre, Region Stockholm, Box 45436

104 31 Stockholm

^3^ Karolinska Institutet, Department of Molecular Medicine and Surgery, Endocrine and Diabetes Unit.

Corresponding author

Marina Taloyan, Associated professor

Division of Family Medicine and Primary Care

Department of Neurobiology, Care Sciences and Society (NVS)

Karolinska Institutet

Alfred Nobels allé 23

Stockholm, SE-14183

Sweden

Phone: 46 737464551

Email: [marina.taloyan@ki.se](mailto:marina.taloyan@ki.se), [marina.taloyan@sll.se](mailto:marina.taloyan@sll.se)

**Key points:**

- In two primary healthcare centres in low socioeconomic status areas of Sweden, 38.4% of individuals had abnormal fasting plasma glucose levels and 41.8% had inadequate serum 25-hydroxy vitamin D levels.
- Those of Middle Eastern descent in Sweden had a much higher prevalence (67.2% vs. 20.5%) and odds (Adjusted odds ratio = 6.7) of having inadequate serum 25-hydroxy vitamin D levels than those of Swedish descent.
- No correlation existed between fasting plasma glucose and serum 25-hydroxy vitamin D levels in those of Swedish descent, and only a weakly positive correlation existed between the levels in those of Middle Eastern descent.

**Abstract**

*Purpose:* To determine fasting plasma glucose and serum 25-hydroxyvitamin D (s-25(OH)D) levels and associations between abnormal fasting plasma glucose levels and inadequate s-25(OH)D levels in individuals of Middle Eastern and Swedish descent.

*Methods:* Observational study with individuals without a diabetes diagnosis, 54.5% of Swedish descent and 45.5% of Middle Eastern descent. In total, 830 participants from two primary healthcare centres in Flemingsberg and Jakobsberg, which are southern and northern suburbs, respectively, of Stockholm, Sweden were included in the study.

*Results:* Prevalence of inadequate s-25(OH)D levels (at or below 50 nmol/L) was 67.2% among those of Middle Eastern descent and 20.5% among those of Swedish descent (*P*<0.001). S-25(OH)D levels correlated weakly positively with fasting plasma glucose levels (ρ=0.20, *P*=0.002) in individuals of Middle Eastern descent. Being of Middle Eastern descent (OR 6.7, 95% CI 4.3-10.4) and having abnormal fasting plasma glucose (OR 1.8, 95% CI 1.2-2.9) were independent predictors of having inadequate s-25(OH)D levels.

*Conclusions:* Healthcare in Sweden should consider testing fasting plasma glucose and s-25(OH)D levels, particularly in individuals of Middle Eastern descent. The unclear relationship between vitamin D and glucose levels warrants investigation.

**Keywords:** 25-hydroxy vitamin D, diabetes, glucose, immigrant, Middle Eastern

# Introduction

Immigration to Sweden from non-European countries has increased in recent decades (1). Many immigrants to Sweden come from Asia, East Africa, and the Middle East. The long, dark winters of Sweden may cause or exacerbate vitamin D deficiency. A 2016 cross-sectional study of Middle Eastern and African immigrants in Umeå, Sweden (latitude 63.8 degrees north) found that 73% of study participants had vitamin D deficiency (2). Furthermore, people from Asia and the Middle East appear to require more sun exposure than people of northern European descent to produce sufficient vitamin D (2, 3). This has been cited as one reason that South Asians in the United Kingdom have lower vitamin D levels than the white population (4). In Sweden, vitamin D deficiency in immigrants from non-European countries has recently become an important public health concern (3, 5).

Known health effects of vitamin D deficiency include fatigue (6), osteoporosis (7), and an increased risk for viral disorders (e.g., COVID-19) because of a reduced immune response (8, 9). Several studies suggest that vitamin D deficiency may also be a risk factor for type 2 diabetes (10-14). Low vitamin D levels are more prevalent in groups of patients with type 2 diabetes than in healthy populations (15). In contrast, a systematic review concluded that recreational sun exposure, known to enhance vitamin D production, reduced the odds of being diagnosed with type 2 diabetes (16). Moreover, there is evidence that vitamin D may play a role in insulin resistance and B-cell dysfunction, both considered important pathophysiological defects in people with type 2 diabetes (12). Various immunological pathways might also play a role in the relationship between vitamin D deficiency and type 2 diabetes (13). Finally, parathyroid hormone, fibroblast growth factor-23, and osteocalcin have each been reported to influence how vitamin D deficiency affects glucose homeostasis (14). Despite all of these findings, the degree to which and the exact mechanisms by which vitamin D affects glucose homeostasis are still unknown.

In 2015, about 450,000 people in Sweden carried a diagnosis of type 2 diabetes; however, many more people in the country may have had undiagnosed diabetes (17). This may have been particularly true of immigrants, as type 2 diabetes is more prevalent in non-European immigrants who live in Sweden or other Nordic countries than in people of Swedish or other Nordic descent (18). Indeed, some immigrant populations are reported to have a 10 times higher risk of diabetes than indigenous Nordic populations. This is particularly relevant because immigrants also tend to have poorer glycaemic control after receiving glucose-lowering therapy than do ethnic Swedes (19).

Thus, a plausible link exists between vitamin D deficiency and type 2 diabetes, and in Sweden the descent of a patient may also play an important role in the prevalence of both disorders. If these links could be better defined, the information could potentially be used by primary healthcare providers and their collaborating providers to better guide their diagnostic evaluations (20). To our knowledge no previous study has actually investigated the relationship between these two disorders in Middle Easterners living in Nordic countries.

To study the possible link between and the potential role of descent in the two disorders, we were interested in looking at a population of patients in Sweden that included both native Swedes and immigrants, involved only those without a previous diagnosis of type 2 diabetes, and received healthcare in low-socioeconomic areas where a higher prevalence of undiagnosed type 2 diabetes may exist (21).

This study aimed to investigate the prevalence of abnormal fasting plasma glucose and s-25(OH)D levels and the association between abnormal fasting plasma glucose levels and inadequate s-25(OH)D levels.

**Patients and methods**

*Study design*

In this observational, cross-sectional study, we analysed data that was collected from March 2014 through March 2016 during the Programme 4D (Four Diagnoses) Type 2 Diabetes Project, a joint undertaking between the Karolinska Institutet and the Stockholm County Council. The Programme 4D Project involved patients at primary healthcare centres in Flemingsberg and Jakobsberg, which are southern and northern suburbs, respectively, of Stockholm, Sweden, and it focused on four prevalent diseases, including type 2 diabetes.

*Study participants*

The healthcare centres at Flemingsberg and Jakobsberg were chosen for the Programme 4D study because both had large proportions of patients who were of foreign origin. Of all individuals visiting the 2 healthcare centres during the period of the study, those 18 years through 74 years of age, and those born either in Turkey, Iran, or Iraq (including the region of Kurdistan) (all hereinafter referred to as of *Middle Eastern descent* or *Middle Eastern individuals*), or in Sweden to parents born in Sweden (hereinafter referred to as of *Swedish descent or Swedish individuals*), were offered inclusion in the study. Patients with a previous diagnosis of diabetes or a mental health disorder were excluded.

Individuals were recruited consecutively in the waiting rooms at the centres, which were open five days a week (closed weekends). Recruitment was performed eight hours a day by assistant nurses (supervised by a physician), who provided a verbal description of the study and an invitation to join the study to individuals who fulfilled the inclusion criteria. Also, written information about the study was available to individuals in the Arabic, Turkish, Kurdish, Persian, English, and Swedish languages. Those individuals opting to participate provided written informed consent for their involvement in the 4D Diabetes study. The Regional Ethical Review Board in Stockholm approved the study (review number 2013/2303-31/3).

*Primary healthcare centres*

Both primary healthcare centres serve large populations of individuals born outside of Sweden, particularly patients originating from Middle Eastern countries, who comprise one of the largest immigrant groups in Sweden. At the time of the study, the centre in Flemingsberg was responsible for 11,145 patients, and the centre in Jakobsberg was responsible for 18,055 patients.

Both centres are located in low-socioeconomic status suburbs of Stockholm, based on the Care Need Index. The Care Need Index in Flemingsberg is 1.97, while that in Jakobsberg is 1.42. The index ranges from below 1 (more affluent) to above 1 (more deprived) (22). It was originally developed to measure the potential workload of Swedish primary healthcare providers and to estimate the risk of patients developing ill health based on socio-economic factors within households in an area, including elderly persons living alone, children under age five, unemployed people, people with low educational status, single parents, high mobility, and foreign-born people. Individuals seeking care at these facilities in low-socioeconomic areas were deemed to be more at risk for pre-diabetes or type 2 diabetes than the general population (21).

*Laboratory sampling*

Study participants were asked to fast for 12 hours prior to their visit for the study. After being interviewed and examined, they provided blood samples to Biobank Stockholm. Samples were transported to the Karolinska Hospital Laboratory within two hours of being provided. Their s-25(OH)D levels were measured using the Electrochemiluminescence immunoassay (ECLIA) method on Roche Cobas® 6000 and Cobas® 8000 modular analysers (Roche Diagnostics GmbH, Mannheim, Germany). This was the standard method used in the laboratory, with a coefficient of variation of 5% and analytic sensitivity of 9 nmol/L. Fasting plasma glucose levels were also measured at the Karolinska University Hospital Laboratory.

*Measures*

For the purposes of this study, participants divided into two groups by their descent, either Middle Eastern or Swedish, as defined above. Age was treated as a continuous variable. Sex was either male or female. Body Mass Index (BMI) was calculated in kg/m^2^ and treated as a continuous variable. Waist circumference was measured in cm, treated as continuous variable, and analysed separately by sex. Patients were considered to be either smokers or non-smokers (including former smokers).

We considered s-25(OH)D levels to be either sufficient (greater than 50 nmol/L) or inadequate (at or below 50 nmol/L), in accordance with the recommendations of the clinical expert group of the Swedish Society of Osteoporosis (Svenska Osteoporossällskapet) (23). We considered fasting plasma glucose levels to be either normal (4.0 mmol/L to 6.0 mmol/L) or abnormal (greater than 6.0 mmol/L).

*Statistical methods*

Descriptive data were reported using means and standard deviations (SD) or medians and interquartile ranges (IQR). The t-test, chi-square test, or Wilcoxon rank sum test were used to analyse differences in the characteristics of participants in the two descent categories. The choice of test was based on whether these characteristics were normally distributed or skewed. The cut-off for significance (two-tailed) was set at 5% (*P* ≤ 0.05) (24). A Spearman correlation coefficient analysis was used to measure the strengths of relationship between the characteristics, with results reported as rho (ρ). Unconditional binary logistical regression was used to estimate the odds ratios (OR) with 95% confidence intervals (CI) for inadequate s-25(OH)D levels, adjusting for explanatory variables such as age, sex, BMI, waist circumference, smoking, and fasting plasma glucose levels. Stata 14 software (Stata Statistical Software: Release 14. StataCorp LLC, College Station, TX, USA) was used for all analyses.

**Results**

*Outcomes*

Of all study participants, 196 (38.4%) had abnormal fasting plasma glucose levels (**Table 2**). Although the mean fasting plasma glucose level was significantly higher in Middle Eastern individuals (5.7 mmol/L) than in Swedish individuals (5.6 mmol/L) (*P* = 0.03), the proportions of Middle Eastern individuals (36.3%) and Swedish individuals (40.9%) with abnormal fasting plasma glucose levels did not differ significantly (*P* = 0.29).

Of all participants, 213 (41.8%) had an inadequate s-25(OH)D level (**Table 2**). The median s-25(OH)D level in Middle Eastern patients (34 nmol/L) was significantly lower than that in Swedish individuals (62 nmol/L) (*P* < 0.001). Also, a significantly larger proportion of Middle Eastern individuals (67.2%) than Swedish individuals (20.5%) had inadequate s-25(OH)D levels (*P* < 0.001). The same pattern was observed when s-25(OH)D level was dichotomized to D vitamin deficiency (<30 nmol/L): 5% of Swedish individuals had deficiency levels while in Middle Eastern the proportion was significantly higher, 37.1% ((*P* < 0.0001).

*Correlations*
For the study population as a whole, s-25(OH)D levels did not correlate with fasting plasma glucose levels (ρ = 0.06, *P* = 0.87) (**Table 3**). On the other hand, the correlation between D vitamin deficiency measured by <30 nmol/L was significantly and positively correlated with fasting plasma glucose levels (ρ = 0.11, *P* = 0.0005). For the Swedish individuals, s-25(OH)D levels also did not correlate with fasting plasma glucose levels (ρ = -0.02, *P* = 0.70). In contrast, for the Middle Eastern individuals, s-25(OH)D levels did demonstrate a significant but weakly positive correlation with fasting plasma glucose levels (ρ = 0.20, *P* = 0.002).

BMI and waist circumference correlated significantly positively with fasting plasma glucose levels in the overall population (ρ = 0.18, *P* = 0.005; ρ = 0.23, *P* < 0.001; respectively), as well as in the Swedish individuals (ρ = 0.18, *P* = 0.003; ρ = 0.18, *P* = 0.003; respectively) and in the Middle Eastern individuals (ρ = 0.15, *P* = 0.04; ρ = 0.14, *P* = 0.03; respectively) (**Table 3**). Also, s-25(OH)D levels showed significant negative correlations with BMI (ρ = -0.20, *P* < 0.001) and waist circumference (ρ = -0.19, *P* = 0.001) in Swedish individuals, but did not show significant correlations with BMI or waist circumference in Middle Eastern patients.

*Predictors of inadequate 25-hydroxy vitamin D levels*

The crude odds of having inadequate s-25(OH)D levels were 8 times higher in those of Middle Eastern descent than in those of Swedish descent (OR = 8.0; 95% CI, 5.3-11.9). When adjusted for age, sex, BMI, smoking, and fasting plasma glucose level, the odds of having inadequate s-25(OH)D levels remained almost 7 times higher for those of Middle Eastern descent than for those of Swedish descent (OR = 6.7; 95% CI, 4.3-10.4). When those of Swedish and Middle Eastern descent were analysed together as a single population, those with abnormal fasting plasma glucose levels had 80% higher odds of having inadequate levels of s-25(OH)D than those with normal fasting plasma glucose levels (OR = 1.8; 95% CI, 1.2-2.9).

**Discussion**

The main results of this study were that individuals of Middle Eastern descent had lower s-25(OH)D levels and had almost seven times higher odds of having inadequate s-25(OH)D levels than those of Swedish descent. A weakly positive correlation between fasting plasma glucose levels and s-25(OH)D levels existed in those with Middle Eastern descent. And finally, regardless descent, those with abnormal fasting plasma glucose levels had an 80% higher risk of having inadequate s-25(OH)D levels than those with normal glucose levels.

The finding on differences in mean fasting plasma glucose between the studied groups is in agreement with our hypothesis that Middle Eastern individuals, who are known to have a higher risk for prediabetes and diabetes, would have higher fasting plasma glucose levels than Swedish individuals, in a low-socioeconomic area. These results are also consistent with the results of studies of populations of patients who already had a diagnosis of type 2 diabetes. In those studies, relative to native populations, immigrants living in northern countries had not only a higher prevalence of type 2 diabetes (18, 19), but also a greater need for support in managing the disease (25). Interestingly, another study revealed a higher prevalence of diabetes in Middle Easterners living in Sweden than Middle Easterners living in the Middle East (26). Some have suggested that the changes in environment and lifestyle that are associated with migration, as well as migration-related mental health issues (e.g., anxiety and sleeping disorders), might explain the higher risk of prediabetes and diabetes in immigrants after moving to Sweden (26, 27). It is also possible that habits involving eating certain ethnic foods, physical inactivity, and tobacco use persist in immigrants and add to this risk (28).

Our findings that s-25(OH)D levels were lower, the prevalence of inadequate s-25(OH)D levels was higher, and adjusted odds of having inadequate s-25(OH)D were higher in those of Middle Eastern descent than in those of Swedish descent are also in line with the findings of others. A variety of authors have reported that vitamin D deficiency was more prevalent in non-European immigrants who lived in northern countries than in native-born residents (2, 4, 5, 29). Specifically, 73% of immigrants from Africa and the Middle East living in the northern Swedish city of Umeå had inadequate vitamin D levels (2). In that study, inadequate vitamin D levels were associated with low fatty fish intake, not travelling abroad, and wearing long-sleeved clothes in summer. In another Swedish study, 73% of Somali-born, dark-skinned woman living in Sweden, but only 1% of native-born, light-skinned Swedish women, had vitamin D deficiency (3). It is possible that features such as darker skin colour and limited skin sun exposure (because of cultural norms dictating that clothing cover most of the body) are responsible for inadequate vitamin D levels in some of these immigrant populations.

Previous studies have suggested a possible link between type 2 diabetes and vitamin D deficiency (10-14). This led us to hypothesize that we might identify a significant negative correlation between fasting plasma glucose and s-25(OH)D levels in the patients we studied. However, we found only a weakly positive correlation between fasting plasma glucose levels and s-25(OH)D levels, and it was observed only in those of Middle Eastern descent, but not in those of Swedish descent or in the study population as a whole.

Consistent with what others have observed, we found that for our entire study population, having abnormal fasting plasma glucose levels predicted an 80% higher risk of having inadequate s-25(OH)D levels. It is possible that we did not observe a more robust and negative correlation because the previous research has focused mainly on the link between type 2 diabetes (rather than abnormal plasma glucose levels) and inadequate vitamin D levels. Nevertheless, the weakly positive or lack of correlation found between fasting plasma glucose and vitamin D levels in our study population who did not have a diagnosis of diabetes suggests that the relationship between vitamin D status and type 2 diabetes risk in a population like ours warrants further investigation.

*Limitations*

A limitation of this study is that we did not have data about other potential predictors of vitamin D deficiency, such as skin colour, clothing habits, sun exposure, vitamin D supplementation, and time spent in countries with sunny climates. Another limitation of the study is that vitamin D levels were measured only once in each participant, just after they were recruited. This meant that we were unable to compare vitamin D levels of single participants during different seasons of the year. This could have changed our results, as previous research in Sweden has demonstrated that mean s-25(OH)D levels increased by 8 nmol/L per month when monitored between April and August (30) Another limitation is that the recruitment of patients in the waiting room of the primary healthcare centres was done when they were there seeking medical attention for active health problems. Thus, the population in our study was not likely representative of otherwise healthy immigrant or native populations in Sweden.

*Conclusions*

Those of Middle Eastern descent in Sweden had a much higher prevalence and odds of experiencing inadequate s-25(OH)D levels than those of Swedish descent. Unexpectedly, a weakly positive correlation was noted between fasting plasma glucose and s-25(OH)D levels, and only in those of Middle Eastern descent. Healthcare providers in Sweden should consider routine testing of fasting plasma glucose and s-25(OH)D levels, particularly in individuals of Middle Eastern descent. The relationship between vitamin D and glucose levels remains unclear and warrants further investigation, in order to better guide the diagnostic evaluations performed by primary health care providers.

**Acknowledgements**

The study was done at Region Stockholm, Academic Primary Healthcare Center and the Division for Family Medicine at the Department of Neurobiology, Care Sciences and Society at Karolinska Institutet. We thank all participants, who contributed voluntarily.

**Conflict of interests**

The authors declared that they have no conflict of interests.

**References**

1. Migrationsverket SMA. Granted resi­dence permits over­views. Available at :<https://www.migrationsverket.se/English/About-the-Migration-Agency/Statistics/Granted-permits-overviews.html>. 2021.

2. Granlund L, Ramnemark A, Andersson C, Lindkvist M, Fharm E, Norberg M. Prevalence of vitamin D deficiency and its association with nutrition, travelling and clothing habits in an immigrant population in Northern Sweden. European journal of clinical nutrition. 2016;70(3):373-9.

3. Demeke T, Osmancevic A, Gillstedt M, Krogstad AL, Angesjö E, Sinclair H, et al. Comorbidity and health-related quality of life in Somali women living in Sweden. Scand J Prim Health Care. 2019;37(2):174-81.

4. Kift R, Berry JL, Vail A, Durkin MT, Rhodes LE, Webb AR. Lifestyle factors including less cutaneous sun exposure contribute to starkly lower vitamin D levels in U.K. South Asians compared with the white population. The British journal of dermatology. 2013;169(6):1272-8.

5. Björk A, Andersson A, Johansson G, Björkegren K, Bardel A, Kristiansson P. Evaluation of sun holiday, diet habits, origin and other factors as determinants of vitamin D status in Swedish primary health care patients: a cross-sectional study with regression analysis of ethnic Swedish and immigrant women. BMC family practice. 2013;14:129.

6. Jarosz AC, El-Sohemy A. Association between Vitamin D Status and Premenstrual Symptoms. J Acad Nutr Diet. 2019;119(1):115-23.

7. Weaver CM, Alexander DD, Boushey CJ, Dawson-Hughes B, Lappe JM, LeBoff MS, et al. Calcium plus vitamin D supplementation and risk of fractures: an updated meta-analysis from the National Osteoporosis Foundation. Osteoporos Int. 2016;27(1):367-76.

8. Grant WB, Lahore H, McDonnell SL, Baggerly CA, French CB, Aliano JL, et al. Evidence that Vitamin D Supplementation Could Reduce Risk of Influenza and COVID-19 Infections and Deaths. Nutrients. 2020;12(4).

9. Mercola J, Grant WB, Wagner CL. Evidence Regarding Vitamin D and Risk of COVID-19 and Its Severity. Nutrients. 2020;12(11).

10. Autier P, Boniol M, Pizot C, Mullie P. Vitamin D status and ill health: a systematic review. The lancet Diabetes & endocrinology. 2014;2(1):76-89.

11. Stadlmayr A, Aigner E, Huber-Schonauer U, Niederseer D, Zwerina J, Husar-Memmer E, et al. Relations of vitamin D status, gender and type 2 diabetes in middle-aged Caucasians. Acta Diabetol. 2015;52(1):39-46.

12. Stivelman E, Retnakaran R. Role of vitamin D in the pathophysiology and treatment of type 2 diabetes. Current diabetes reviews. 2012;8(1):42-7.

13. Harinarayan CV. Vitamin D and diabetes mellitus. Hormones. 2014;13(2):163-81.

14. Thomas GN, Scragg R, Jiang CQ, Chan W, Marz W, Pilz S, et al. Hyperglycaemia and vitamin D: a systematic overview. Current diabetes reviews. 2012;8(1):18-31.

15. Pannu PK, Piers LS, Soares MJ, Zhao Y, Ansari Z. Vitamin D status is inversely associated with markers of risk for type 2 diabetes: A population based study in Victoria, Australia. PloS one. 2017;12(6):e0178825.

16. Shore-Lorenti C, Brennan SL, Sanders KM, Neale RE, Lucas RM, Ebeling PR. Shining the light on Sunshine: a systematic review of the influence of sun exposure on type 2 diabetes mellitus-related outcomes. Clinical endocrinology. 2014;81(6):799-811.

17. Diabetesförbundet. Diabetes i siffror. Stockholm: Diabetesförbundet.; 2016 [

18. Wändell PE, Carlsson A, Steiner KH. Prevalence of diabetes among immigrants in the Nordic countries. Current diabetes reviews. 2010;6(2):126-33.

19. Rawshani A, Svensson AM, Rosengren A, Zethelius B, Eliasson B, Gudbjörnsdottir S. Impact of ethnicity on progress of glycaemic control in 131,935 newly diagnosed patients with type 2 diabetes: a nationwide observational study from the Swedish National Diabetes Register. BMJ open. 2015;5(6):e007599.

20. Sorensen M, Groven KS, Gjelsvik B, Almendingen K, Garnweidner-Holme L. The roles of healthcare professionals in diabetes care: a qualitative study in Norwegian general practice. Scand J Prim Health Care. 2020;38(1):12-23.

21. Agardh E, Allebeck P, Hallqvist J, Moradi T, Sidorchuk A. Type 2 diabetes incidence and socio-economic position: a systematic review and meta-analysis. Int J Epidemiol. 2011;40(3):804-18.

22. Sundquist K, Malmstrom M, Johansson SE, Sundquist J. Care Need Index, a useful tool for the distribution of primary health care resources. Journal of epidemiology and community health. 2003;57(5):347-52.

23. Lorentzon M AK, Mellström D, Landin-Wilhelmsen K, Pernow Y, Bergström I ea. [D-vitaminbehandling och skeletthälsa-svenska riktlinjer behövs. Rekommendationer fran Svenska osteoporossällskapets kliniska expertgrupp]. Lakartidningen. 2014;111(37):1508-10.

24. Hosmer D. Applied Logistic Regression.; 2002. Inc neeNYJWS, editor2002.

25. Abuelmagd W, Osman BB, Hakonsen H, Jenum AK, Toverud EL. Experiences of Kurdish immigrants with the management of type 2 diabetes: a qualitative study from Norway. Scand J Prim Health Care. 2019;37(3):345-52.

26. Steiner KH, Johansson SE, Sundquist J, Wandell PE. Self-reported anxiety, sleeping problems and pain among Turkish-born immigrants in Sweden. Ethn Health. 2007;12(4):363-79.

27. Taloyan M, Johansson LM, Johansson SE, Sundquist J, Kocturk TO. Poor self-reported health and sleeping difficulties among Kurdish immigrant men in Sweden. Transcult Psychiatry. 2006;43(3):445-61.

28. Golden SH, Yajnik C, Phatak S, Hanson RL, Knowler WC. Racial/ethnic differences in the burden of type 2 diabetes over the life course: a focus on the USA and India. Diabetologia. 2019;62(10):1751-60.

29. Palacios C, Gonzalez L. Is vitamin D deficiency a major global public health problem? The Journal of steroid biochemistry and molecular biology. 2014;144 Pt A:138-45.

30. Klingberg E, Oleröd G, Konar J, Petzold M, Hammarsten O. Seasonal variations in serum 25-hydroxy vitamin D levels in a Swedish cohort. Endocrine. 2015;49(3):800-8.

**Tables**

**Table 1.** Demographic and clinical characteristics of study participants (N = 510)

| ***Characteristics*** | **Study Participants** | | | **P-value** |
| --- | --- | --- | --- | --- |
|  | **All** | **Swedish Descent^a^** | **Middle Eastern Descent^b^** |  |
| **Total**, n (%) | 510 (100) | 278 (100) | 232 (100) | - |
| **Female**, n (%) | 291 (57.1) | 156 (56.1) | 135 (58.2) | 0.64^c^ |
| **Age**, mean (SD), *years* | 51.0 (14.5) | 55.8 (15.0) | 45.3 (11.7) | <0.001^d^ |
| **BMI**, mean (SD), *kg/m^2^* | 28.5 (6.2) | 28.2 (5.8) | 28.9 (6.8) | 0.21^d^ |
| **Waist circumference, male,** mean (SD), *cm* | 102.7 (14.0) | 104.8 (13.5) | 100.1 (14.2) | 0.01^d^ |
| **Waist circumference, female,** mean (SD), *cm* | 94.2 (13.4) | 95.8 (14.0) | 92.8 (12.6) | 0.05^d^ |
| **Smoking,** n (%) | 96 (18.9) | 37 (38.5) | 59 (61.5) | <0.001^c^ |

^a^ Born in Sweden to parents born in Sweden.

^b^ Born in or one parent born in Turkey, Iran, or Iraq [including region of Kurdistan].

^c^ Based on the chi-square test.

^d^ Based on t-test.

Abbreviations: SD, standard deviation; BMI, body mass index; s-25(OH)D, serum 25-hydroxy vitamin D

**Table 2.** Fasting plasma glucose and serum 25-hydroxy vitamin D levels of study participants (N = 510)

| ***Outcomes*** | **Study Participants** | | | **P-value** |
| --- | --- | --- | --- | --- |
|  | **All** | **Swedish Descent^a^** | **Middle Eastern Descent^b^** |  |
| **Total**, n (%) | 510 (100) | 278 (100) | 232 (100) | - |
| **Fasting plasma glucose level,** mean (SD), *mmol/L* | 5.7 (0.8) | 5.7 (0.5) | 5.6 (0.5) | 0.03^d^ |
| **Abnormal (**> 6.0 mmol/L**) fasting plasma glucose**, n (%) | 196 (38.4) | 101 (36.3) | 95 (40.9) | 0.29^c^ |
| **s-25(OH)D level**, median (IQR), *nmol/L* | 46 (28-68) | 62 (50-86) | 34 (21-47) | <0.001^e^ |
| **Inadequate (**≤ 50 nmol/L**)**  **s-25(OH)D**, n (%) | 213 (41.8) | 57 (20.5) | 156 (67.2) | <0.001^c^ |
| **Deficiency** (<30 nmol/L)  s-25(OH)D, n (%) | 100 (19.6) | 14 (5.0) | 86 (37.1) | <0.0001^c^ |

^a^ Born in Sweden to parents born in Sweden.

^b^ Born in or one parent born in Turkey, Iran, or Iraq [including region of Kurdistan].

^c^ Based on the chi-square test.

^d^ Based on t-test.

^e^ Based on Wilcoxon rank sum test.

Abbreviations: SD, standard deviation; s-25(OH)D, serum 25-hydroxy vitamin D; IQR, interquartile range

**Table 3.**  Correlations in Individuals of Swedish and Middle Eastern descent between fasting plasma glucose, serum 25-hydroxy vitamin D, age, and weight status (N = 510)

| ***Characteristics*** | **Study Participants** | | | | | | | | | | | |
| --- | --- | --- | --- | --- | --- | --- | --- | --- | --- | --- | --- | --- |
|  | **All** | | | | **Swedish Descent^a^** | | | | **Middle Eastern Descent^b^** | | | |
|  | **FPG** | | **s-25(OH)D** | | **FPG** | | **s-25(OH)D** | | **FPG** | | **s-25(OH)D** | |
|  | ρ^c^ | *P*-value | ρ^c^ | *P*-value | ρ^c^ | *P*-value | ρ^c^ | *P*-value | ρ^c^ | *P*-value | ρ^c^ | *P*-value |
| s-25(OH)D level | 0.06 | 0.87 | - | - | -0.02 | 0.70 | - | - | **0.20** | 0.002 | - | - |
| D vitamin deficiency (<30nmol/L) | **0.11** | 0.0005 | - | - | 0.00 | 0.96 | - | - | 0.04 | 0.54 | - | - |
| Age | **0.17** | <0.001 | 0.32 | <0.001 | **0.13** | 0.03 | 0.05 | 0.36 | 0.07 | 0.26 | 0.09 | 0.20 |
| Body mass index | **0.18** | 0.005 | **-0.13** | 0.007 | **0.18** | 0.003 | **-0.20** | <0.001 | **0.15** | 0.04 | 0.01 | 0.94 |
| Waist circumference | **0.23** | <0.001 | -0.04 | 0.27 | **0.18** | 0.003 | **-0.19** | 0.001 | **0.14** | 0.03 | -0.03 | 0.63 |

^a^ Born in Sweden to parents born in Sweden.

^b^ Born or one parent born in Turkey, Iran, or Iraq [including region of Kurdistan].

^c^ rho (ρ) is the correlation coefficient based on Spearman analysis, used to measure the strength of the relationship.

Abbreviations: FPG, fasting plasma glucose; s-25(OH)D, serum 25-hydroxy vitamin D
